# Radiofrequency ablation versus ^125^I-seed brachytherapy for painful metastases involving the bone

**DOI:** 10.18632/oncotarget.11983

**Published:** 2016-09-12

**Authors:** Dechao Jiao, Gang Wu, Jianzhuang Ren, Xinwei Han

**Affiliations:** ^1^ Department of Interventional Radiology, The First Affiliated Hospital of Zhengzhou University, Zhengzhou, Henan, People’s Republic of China

**Keywords:** radiofrequency ablation, ^125^I-seed, brachytherapy, bone, metastases

## Abstract

This retrospective study aimed to demonstrate and compare the safety and effectiveness of computed tomography-guided radiofrequency ablation (RFA) and ^125^I-seed brachytherapy for painful bone metastases after failure of external beam radiotherapy (EBRT). From June 2013 to October 2015, 79 patients with moderate-to-severe pain caused by metastatic bone lesions who underwent either RFA (n = 41) or ^125^I-seed brachytherapy (n = 38) were enrolled. Pain in patients was measured using the brief pain inventory (BPI) before treatment, 1 week after treatment, and 3 months after treatment. Response rates were assessed by measuring the changes in pain and incorporation of changes in the analgesic requirements. At baseline, 1 week, and 3 months, the mean worst pain scores of BPI were 7.8, 5.4, and 2.7, respectively, for the RFA group and 7.7, 6.1, and 2.8, respectively, for the brachytherapy group. At 1 week, the complete and partial response rates were 12% and 59%, respectively, in the RFA group compared with 3% and 45%, respectively, in the brachytherapy group. At 3 months, the complete and partial response rates were 23% and 58%, respectively, in the RFA group compared with 24% and 52% in the brachytherapy group (p = 0.95). The response rates in the RFA group were significantly higher than those in the brachytherapy group at 1 week (p = 0.32), but comparable at 3 weeks (p = 0.95). Both groups had low rates of complications and no treatment-related mortality. In conclusion, the short-term curative efficiency of RFA was better than that of brachytherapy, but the log-term efficiency of both treatments was equal.

## INTRODUCTION

Bone metastasis is the primary leading cause of morbidity in patients with advanced cancer. The clinical symptoms include pain, dysfunction, pathological fracture, and decreased mobility, all of which can seriously affect patients’ quality of life [1, 2]. Tumour invasion or compression of the periosteum or adjacent neural structures can lead to local or radiating pain. Approximately 70% of patients with advanced cancer experience bone pain, which could impair sleep, diet, emotion, and daily activities if left untreated [3, 4]. The current primary treatments available for patients with bone metastases are palliative. For diffuse metastases, a systemic approach comprising chemotherapy, hormonal therapy, radiopharmaceuticals, and bisphosphonates is recommended. External beam radiotherapy (EBRT) is the preferred strategy for localised painful bone metastases, but > 30% of patients do not achieve pain relief, and nearly 50% of patients have recurrent pain after EBRT [5–7].

Considering the poor quality of life and short life expectancy in these patients, a minimally invasive interventional technique is needed, and new therapeutic approaches that offer a curative option have been gaining attention during the last two decades. The new interventional techniques rely on imaging guidance to direct vessels of the metastatic tissue, along with different therapeutic procedures such as radiofrequency ablation, cryoablation, microwave ablation, and radioactive seeds implantation [8–10].

A new interventional approach known as radiofrequency ablation (RFA) utilises a high-frequency alternating current that is released from a needle electrode into the surrounding tissue, which causes frictional heating and necrosis of the tumour tissue. Two multicentric studies previously evaluated the effectiveness of RFA in patients who were unresponsive to standard treatments and reported significantly reduced local pain [11, 12]. However, RFA has a critical defect: the ablation margin cannot be visualised even with computed tomography (CT) monitoring, due to which adjacent crucial organs may be harmed or undertreated.

Patients may not respond to EBRT due to the radiation insensitivity of the neoplasm and dose limitation to protect the adjacent normal tissues. Another new therapeutic approach—percutaneous iodine-125 (^125^I)-seed brachytherapy—may resolve this problem by delivering a high dose of radiation directly to the tumour-bearing region while sparing adjacent healthy tissue [13]. Furthermore, patients undergoing ^125^I-seed brachytherapy do not experience increased pain during or after the procedure. However, we found only a few reports on the use of ^125^I seed brachytherapy for bone metastases in the literature.

Considering that both RFA and ^125^I-seed implantation help reduce pain, the aim of this study was to demonstrate and compare the safety and effectiveness of CT-guided ^125^I-seed implantation and RFA for painful bone metastases after failure of EBRT.

## RESULTS

### Baseline characteristics of patients in the RFA and brachytherapy groups

The baseline patient demographics and tumour characteristics of the patients in the two groups are presented in Table 1. The final study group comprised 41 patients (median age, 51 years; age range, 18-76 years; 25 men and 16 women) in the RFA group and 38 patients (median age, 50 years; age range, 27-81 years; 22 men and 16 women) in the brachytherapy group. In the RFA group, 21 patients (49%) had received chemotherapy, 14 patients (37%) had received bisphosphonates, and 32 patients (84%) had received opioid analgesics for the ostealgia due to metastatic cancer before entering the study. In the brachytherapy group, 20 patients (49%) had received chemotherapy, 14 patients (37%) had received bisphosphonates, and 32 patients (84%) had received opioid analgesics before entering the study. In both groups, the most common primary types of cancer were carcinoma of the lungs and liver. The most common site of ostealgia was the pelvis in the RFA group and the vertebrae in the brachytherapy group. The patients in the RFA group had a larger tumour size than those in the brachytherapy group (p < 0.001). Furthermore, in the RFA group, a single lesion was treated in 37 patients and two lesions were treated in 4 patients, whereas in the brachytherapy group, a single lesion was treated in 36 patients and two lesions were treated in 4 patients.

**Table 1:** Baseline characteristics of the study patients

	Radiofrequency ablation	I125 seed brachytherapy
No. of patients	41	38
Age, years		
Median	51	50
Range	18-76	27-81
Sex		
Men	25 (61)	22 (58)
Women	16 (39)	16 (42)
Primary cancer site		
Lung	13 (32)	11(29)
Liver	13 (32)	8 (21)
Colon/rectum	4 (10)	5 (13)
Kidney	4 (10)	3 (8)
Breast	2 (5)	3 (8)
Prostate	0 (0)	2 (5)
Other	4 (10)	6 (16)
Sites of painful bone lesion		
Pelvis	19 (46)	7 (18)
Sacrum	7 (17)	5 (13)
Rib/sternum	6 (15)	7 (18)
Vertebrae	4 (10)	13 (34)
Scapula	3 (7)	3 (8)
Extremity	2 (5)	4 (11)
Previous treatment		
Chemotherapy	21 (49)	20 (53)
Bisphosphonates	16 (39)	13 (34)
Opioid analgesics	36 (88)	33 (87)
Tumor size, cm (longest diameter)		
Mean	5.7	3.6
Range	1.7-13.2	1.5-7.1
Metastases numbers		
1	37 (90)	36 (95)
2	4 (10)	2 (5)
Type of bone metastases		
Osteolytic	35 (85)	27 (71)
Osteoplastic	0 (0)	6 (16)
Mixed	6 (15)	5 (13)

### Patients’ responses to pain treatment

During the treatment, patients in both groups experienced a large reduction in their worst pain, average pain, and pain interference and showed significant improvements in pain relief after 1 week and 3 months of treatment (Table 2). Prior to RFA, the mean scores of BPI were 7.8 for the worst pain, 5.7 for the mean pain, and 6.4 for mean pain interference with daily life. One week after RFA, the scores reduced to 5.4 (p < 0.001), 3.6 (p < 0.001), and 4.7 (p < 0.001), and after 3 months, they reduced to 2.7 (p < 0.001), 1.7 (p < 0.001), and 2.5 (p = 0.005) for the worst pain, the mean pain, and mean pain interference with daily life, respectively. After RFA, pain relief improved from 45% at baseline to 71% at 1 week (p < 0.0001) and 82% at 3 months (p = 0.002).

**Table 2:** Brief Pain Inventory-Short Form mean pain scores and opioid requirements at baseline and after treatment

	Baseline	Week 1	Month 3
Worst pain (0–10)			
RFA	7.8	5.4	2.7
I125 seed	7.7	6.1	2.8
Average pain (0–10)			
RFA	5.7	3.6	1.7
I125 seed	5.6	4.4	1.8
Pain interference (0–100)			
RFA	6.4	4.7	2.5
I125 seed	6.4	5.4	2.7
Pain relief (0–10)			
RFA	45	71	82
I125 seed	47	64	82
Morphine-equivalent dose			
RFA	103	107	42
I125 seed	101	95	46

Before ^125^I-seed implantation, the mean BPI scores were 7.7 for the worst pain, 5.6 for mean pain, and 6.4 for mean pain interference with daily life. These scores reduced to 6.1 (p < 0.001), 4.4 (p < 0.001), and 5.4 (p < 0.001) at 1 week after the ^125^I-seed implantation and to 2.8 (p < 0.001), 1.8 (p < 0.001), and 2.7 (p = 0.005) at 3 months after implantation, respectively. Pain relief after ^125^I-seed implantation improved from 47% at baseline to 64% at 1 week (p < 0.0001) and 82% at 3 months (p = 0.002).

### Local control efficacy and complications

The BPI form was completed by all 79 patients 1 week after the treatment (Table 3). Complete response was observed in 12% of patients (5 patients) in the RFA group and 3% of patients (1 patient) in the brachytherapy group. Further, partial response was observed in 59% of patients (24 patients) in the RFA group and 45% of patients (17 patients) in the brachytherapy group. The 3-month BPI assessment was completed for 51 patients (26 in the RFA group and 25 in the brachytherapy group) of the 79 patients (Table 3). The reasons for missing BPI data at 3 months included patients’ death (14 patients), loss to follow-up (8 patients), refusal or too ill to complete (5 patients), and initiation of surgical resection (n = 1). The complete response and partial response rates for the 26 patients in the RFA group were 23% (6 patients) and 58% (15 patients), respectively, and for the 25 patients in the brachytherapy group were 24% (6 patients) and 52% (13 patients) respectively (p = 0.6).

**Table 3:** Response to treatment according to the Brief Pain Inventory score and daily oral morphine equivalent

	1 Week		3 months	
	Radiofrequency ablation (n = 41)	Brachytherapy (n = 38)	Radiofrequency ablation (n = 26)	Brachytherapy (n = 25)
Overall response	29 (71%)	18 (47%)	21 (81%)	19 (76%)
Complete response	5 (12%)	1 (3%)	6 (23%)	6 (24%)
Partial response	24 (59%)	17 (45%)	15 (58 %)	13 (52%)
Indeterminate response	12 (29%)	19 (50%)	5 (21%)	4 (16%)
Pain progression	1 (2%)	1 (3%)	1 (4%)	2 (8%)

In the RFA group, 2 of 41 patients (5%) experienced a major complication: One patient experienced a Grade 2 skin burn at the grounding pad site and recovered after conservative treatment, whereas the other patient experienced a second-degree pleural effusion following RFA of rib metastasis. No treatment-related mortality occurred in the RFA group. In the brachytherapy group, three complications were observed: Two patients developed Grade 1 local skin reaction, and one patient developed Grade 2 myelosuppression with iliac metastases, but returned to the normal state within 3 months.

## DISCUSSION

Bone metastasis is the most common source of cancer-related pain and is difficult to manage [1, 2]. EBRT is considered the standard care for management of localised uncomplicated painful bony metastases, and local field irradiation is a well-recognised method for palliation of ostealgia. In 2005, the Radiation Therapy Oncology Group (RTOG) conducted a prospective phase III randomised trial of palliative radiation therapy for patients who were diagnosed with painful osseous metastases and had moderate-to-severe pain [7]. An overall response was observed in 66% of the patients, and a complete response was observed in 17% patients at 3 months after randomisation. Although similar results have been reported in other randomised trials, the definitions of ‘response’ varied among the studies [6, 14–19]. A consensus meeting on the treatment for bone metastases recommended inclusion of changes in patient pain and the incorporation of changes in analgesic requirements in the response criteria, as used in the current study [20]. The potential reason for the EBRT failure might be the limitation of radiation dose. The surrounding normal tissue of bone lesions is inevitably radioactively damaged after EBRT. Therefore, the limited dose of EBRT by the tolerance of the normal tissue may cause incomplete killing of tumor cells, especially for radiation insensitive tumor cells. Furthermore, sublethal damaged tumor cells could self-repair and proliferate thereafter.

Image-guided RFA is now an optional treatment for ostealgia due to metastasis of carcinoma after failure of conventional therapies. According to a multicentre study involving 43 patients with ostealgia, RFA was an effective and safe treatment for pain reduction and decrease with the use of opioids. Furthermore, this trial reported that the mean score of BPI for the worst pain was 7.9 before treatment, 5.8 after 1 week, and 3.0 after 12 weeks of treatment [11]. Similarly, another multicentre study used a modified Memorial Pain Assessment Card for assessment of pain and concluded that RFA could be used to safely palliate ostealgia [12]. Our findings confirmed the efficiency and safety of RFA, which is consistent with the findings of the abovementioned studies.

Although RFA is an effective treatment for ostealgia, it has some drawbacks. First, since the margin of the ablation is often unclear during the operation, the lesion chosen should be away from vital organs and structures (i.e. spinal cord, major motor nerves, artery, or bowel) to avoid injury or inefficient therapy. Second, due to low conductivity and relative permittivity of bone, RFA requires the treated lesions to be lytic or have a lytic component, which is not suitable for osteoplastic lesions. Third, as there can be severe pain during RFA, some patients need to be administered general anaesthesia, which might not be appropriate for high-risk patients.

In this study, we reported a highly significant alleviation in pain and improvement in the quality of life following ^125^I-seed implantation for painful metastases involving bone. Our findings are similar to those reported by Feng et al., with an overall response rate of 58% by 1 week and 82% by 12 weeks after implantation. Patients in the brachytherapy group achieved pain relief later than those in the RFA group, with a 1.7-point reduction in the mean worst pain in the brachytherapy group and a 2.5-point reduction in the RFA group by 1 week after implantation. Similarly, the overall response in the RFA group (71%) was significantly better than that in the brachytherapy group (47%). Furthermore, the brachytherapy group showed slower efficiency in the treatment for ostealgia since the implanted seed needed time to release a sufficient radiation dose to cure the lesions. After 3 months, patients in the RFA group experienced a 4.6-point reduction in the mean worst pain, while patients in the brachytherapy group experienced a 4.4-point reduction. The overall response rate between the two groups did not differ significantly (81% in the RFA group and 76% in the brachytherapy group), which indicated that although RFA had significant superiority in the short-term, its long-time efficiency was equal to that of ^125^I-seed brachytherapy. The most common site of ostealgia was the vertebrae in the brachytherapy group (34%), but not in the RFA group (10%). This could be due to the operators’ consideration for injury to the spinal cord and major nerve during RFA, because of which they may have preferred ^125^I-seed implantation. The success of ^125^I-seed brachytherapy for a tumour depends on the precise placement of radioactive seeds [21, 22]. The American Brachytherapy Society's “dual 90” principle stated that the cure for cancer required 90% of the tumour volume to obtain 90% of the prescription dose [23]. Since the radiation released by the ^125^I-seed decreases with distance, we found that TPS could help peripheral tumour doses to achieve the matched peripheral dose (MPD) of 100-120 Gy and ensure that > 95% of the tumour volume achieves 100% of the prescription dose^24^. Therefore, the target tumour can receive the adequate dose without extra radiation to the surrounding normal tissues.

In the RFA group, all patients were diagnosed with osteolytic metastases or mixed metastases but without osteoblastic metastases. Seed-implantation brachytherapy can be used as a therapy for osteoblastic metastases in addition to osteolytic metastases. The largest tumour was 13.2 cm in size in the RFA group and 7.1cm in the brachytherapy group. For large tumours, it is difficult to ensure ^125^I-seed distribution in accordance with the TPS. Therefore, for such tumours, we performed RFA in safe areas and supplemented it with ^125^I-seed brachytherapy adjacent to important structures.

In our study, both treatment groups had low rates of complications, and there was no treatment-related mortality. The rate of complications was 5% (2/41 patients) in the RFA group, and 7.9% (3/38 patients) in the brachytherapy group, which is comparable with that of other studies [10, 11, 12]. All patients undergoing major complications fully recovered after symptomatic treatment and none of them had any serious sequelae. These results suggest that both the treatment regimens were safe for bone metastases.

Despite our important findings, our study had two main limitations: the sample size was small and the duration of follow-up was short. Therefore, multicenter randomised controlled trails should be carried out in the future to assess the curative efficiency of RFA and ^125^I-seed implantation brachytherapy for ostealgia due to cancer metastases. Other minor limitations of the study included the heterogeneous population of enrolled patients in terms of sites, size of the metastasis, and presence of primary treated tumours in patients. Although breast and prostate carcinomas are the most common cancers accompanied with bone metastases, the most common primary carcinomas in our study were lung and liver cancers, which may be due to the widespread occurrence of lung and liver cancers in China. In addition, breast and prostate carcinomas are sensitive to radiotherapy, and most of the patients with bone metastases could control the pain well following EBRT, discounting the need for advanced ^125^I-seed implantation brachytherapy.

In conclusion, the short-term curative efficiency of RFA was better than that of brachytherapy, but the log-term efficiency of both treatments was equal. Our results suggest that ^125^I seed implantation could be considered an alternative treatment regimen in patients with painful bone metastasis, especially for lesions close to vital organs and structures. However, our findings should be confirmed in prospective randomised controlled trials.

## MATERIALS AND METHODS

This study was approved by the local institutional review board, and informed consent was obtained from all participants. Patients’ medical records were reviewed, and the following data were collected and analysed: demographic and clinical data, tumour features, and information about pain.

### Patients

Seventy-nine patients with moderate-to-severe pain caused by metastatic bone lesions who underwent RFA or ^125^I-seed brachytherapy from June 2013 to October 2015 were enrolled in this retrospective study. All patients had a previous history of EBRT at the proposed site and other conventional therapies such as opioid agents and chemotherapy for the refractory sites. The proposed site should qualify for the percutaneous approach, i.e., the RFA electrode and seed-implantation applicators should be safely placed in the location without significant harm to normal tissues.

All patients in our study met the following criteria: (1) pathologically confirmed malignant disease and radiographic evidence of bone metastasis; (2) up to two separate sites of painful metastases; (3) at least four points for highest pain at the metastatic site over the past 24 h, as assessed by the Brief Pain Inventory (BPI); (4) lesions unresponsive to chemotherapy or radiotherapy for at least 4 weeks before the current study; (5) expected life span ≥ 3 months; (6) Karnofsky performance status scores ≥ 50; (7) international normalised ratio ≤ 1.5 and platelet count > 50,000/μL; (8) ability to tolerate RFA or ^125^I-seed brachytherapy with CT imaging; and (9) absence of spinal-cord compression or impending fracture. After excluding patients who failed to appear for follow-up (n = 5) or receive cementoplasty (n = 3), data of the remaining 46 patients were analysed.

### Pre-treatment preparation

Before treatment, the pain resulting from metastatic disease was assessed by BPI. The use of opioid analgesic medication was recorded. All patients underwent pre-procedural contrast-enhanced CT to allow evaluation of the lesions’ location, size, and radiological characteristics. Physical examination was performed by the oncologist with radiologist's assistance. Within 1 week before the implantation procedure, complete blood count and prothrombin time measurement were performed.

### Radiofrequency ablation

**Figure 1:**
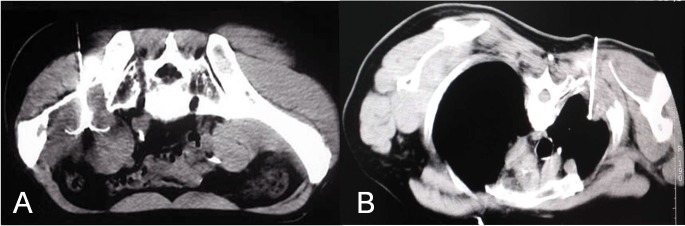
Computed tomography images showing the percutaneous placement of the radiofrequency ablation electrode **A.** Image of a 57-year-old man with metastatic hepatocellular carcinoma involving the right ilium. **B.** Image of a 67-year-old man with metastatic non-small-cell lung carcinoma involving the rib.

Under general anaesthesia or moderate sedation, patients were treated with local anaesthesia involving 1% lidocaine intradermally and periosteumally. Two dispersive electrode-grounding pads were positioned on the patients’ thighs. A 14-gauge coaxial needle (Ackerman, Cook, Bloomington, IN) was placed in the lesion for bone biopsy if the compact bone substance was intact. After the core and inner trephine needle was removed, the electrode passed through the cannula into the lesions. For tumours destroying the bone cortex, the RF electrode directly passed through and into the metastasis. The RFA electrode (StarBurst XL, Model 1500X; Angio Dynamics, Queensbury, NY; Cool-tip RFA system; Valleylab, Boulder, CO) was inserted into the lesion for treatment under CT guidance at a predetermined angle and depth (Figure 1). Power settings and ablation times were selected on the basis of standard recommendations by the manufacturer. For eradication of the entire tumour, overlapping techniques were adopted for tumors measuring more than 3 cm in the longest dimension.

### ^125^I-seed implant brachytherapy

Before ^125^I-seed brachytherapy, 5-mm axial enhanced CT images were obtained from all patients. The Brachytherapy Treatment Planning System (TPS) (Beijing Atom and High Technique Industries, Inc., Beijing, China) was used to design implants according to the CT images. On the basis of the latest CT scans, the gross tumour volume was precisely identified. Oncologists depicted the prescribed dose target volume that covered the lesion with a 0.5-1 cm safe margin. The specified dose of radiation was 100-120 Gy, which changed according to adjacent structures and the previous radiation dose. On the basis of three orthogonal diameters for the target tumour and an average prescribed MPD of 110 Gy, the TPS created a dose-volume histogram and isodose curves for different percentages and calculated the position for implantation needles and the number for implanted seeds.

**Figure 2:**
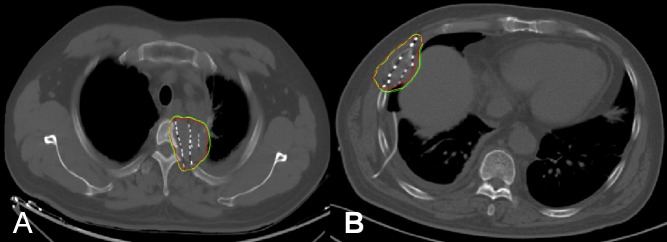
Administration of ^125^I-seed brachytherapy The isodose curve after seed implantation on CT scan. Red lines represent the contour of the tumour. The yellow and green curves are isodose lines of D100 (120 Gy) and D90 (108 Gy), respectively. A. Image of a 61-year-old man with metastatic esophageal cancer involving the thoracic vertebra. B. Image of a 48-year-old man with metastatic non-small-cell lung carcinoma involving the rib.

The ^125^I-seed implantation was performed under moderate sedation and routine CT guidance. After local anaesthesia with 1% lidocaine, an 18-G implantation needle (Yunke Pharmaceutical Limited Liability Company, Chengdu, China) was inserted into the tumour's farthest edge, but was maintained approximately 5 mm within the border. Thereafter, a clip or turntable implant gun (Yunke Pharmaceutical Limited Liability Company, Chengdu, China) was attached to the implantation needle. On retracting the needle, particles were released from the deep to the shallow end. After the implantation, a CT scan was performed to detect any postoperative complications such as bleeding. To verify the position and intensity of the ^125^I seeds, the last scan image was reviewed according to the TPS (Figure 2). If a lesion presented with insufficient radioactivity, the procedure was repeated for additional ^125^I-seed implantation.

### Post-treatment patient assessment

Post-treatment assessment was conducted for each patient. BPI was used to evaluate the severity of pain. The patients completed the BPI with assistance from a study coordinator majoring in focal painful metastasis. When multiple metastases were treated in one patient, the response was recorded for the most painful treated area. BPI was performed at 1 week and 3 months after treatment. Analgesic use was precisely recorded during each interview and translated into a morphine-equivalent dose [24]. Each patient underwent a contrast-enhanced CT or positron emission tomography-CT examination for the treated area in 3-5 weeks after therapy. A complete response was defined as reduction of the BPI worst-pain score to zero at the treated site, with a stable or daily reducing oral dose of equivalent morphine. A partial response was defined as a reduction in the worst-pain score by ≥ 2 points for the treated site without an increase in the daily oral dose of equivalent morphine, or as a ≥ 25% reduction in analgesic use from the baseline without an increase in pain. Pain progression was defined as an increase in the pain score by ≥ 2 points above the baseline for the treated site along with a stable analgesic intake, or as an increase in the daily oral dose of equivalent morphine of least 25%, with a stable worst-pain score or 1 point bove the baseline. An indeterminate response was defined as any response that did not conform to the definitions of complete response, partial response, or pain progression [20]. The overall response rate was the sum of the complete response rate and partial response rate.

### Statistical analysis

The worst pain, average pain, pain interference, pain relief, and morphine-equivalent dose were analysed using paired t-tests at each follow-up, supplemented by repeated measurement analysis of correlated variance. We analysed the proportion of complete response, partial pain response, indeterminate response, and pain progression of the two groups using a Kruskal Wallis H-test. The statistical software package IBM SPSS Statistics 20.0 (IBM Co, Armonk, NY) was used for statistical analyses. Values of p < 0.05 were considered statistically significant.

## References

[1] Coleman RE (2006). Clinical features of metastatic bone disease and risk of skeletal morbidity. Clin Cancer Res.

[2] Nielsen OS, Munro AJ, Tannock IF (1991). Bone metastases: pathophysiology and management policy. J Clin Oncol.

[3] Falk S, Dickenson AH (2014). Pain and nociception: mechanisms of cancer-induced bone pain. J Clin Oncol.

[4] Mercadante S (1997). Malignant bone pain: pathophysiology and treatment. Pain.

[5] Rades D, Schild SE, Abrahm JL (2010). Treatment of painful bone metastases. Nat Rev Clin Oncol.

[6] Jeremic B, Shibamoto Y, Acimovic L, Milicic B, Milisavljevic S, Nikolic N, Aleksandrovic J, Igrutinovic I (1998). A randomized trial of three single-dose radiation therapy regimens in the treatment of metastatic bone pain. Int J Radiat Oncol Biol Phys.

[7] Hartsell WF, Scott CB, Bruner DW, Scarantino CW, Ivker RA, Roach MR, Suh JH, Demas WF, Movsas B, Petersen IA, Konski AA, Cleeland CS, Janjan NA, DeSilvio M (2005). Randomized trial of short- versus long-course radiotherapy for palliation of painful bone metastases. J Natl Cancer Inst.

[8] Rosenthal D, Callstrom MR (2012). Critical review and state of the art in interventional oncology: benign and metastatic disease involving bone. Radiology.

[9] Botsa E, Mylona S, Koutsogiannis I, Koundouraki A, Thanos L (2014). CT image guided thermal ablation techniques for palliation of painful bone metastases. Ann Palliat Med.

[10] Feng S, Wang L, Xiao Z, Maharjan R, Chuanxing L, Fujun Z, Jinhua H, Peihong W (2015). ^125^I Seed Implant Brachytherapy for. Painful Bone Metastases After Failure of External Beam Radiation Therapy. Medicine (Baltimore).

[11] Goetz MP, Callstrom MR, Charboneau JW, Farrell MA, Maus TP, Welch TJ, Wong GY, Sloan JA, Novotny PJ, Petersen IA, Beres RA, Regge D, Capanna R, Saker MB, Gronemeyer DH, Gevargez A (2004). Percutaneous image-guided radiofrequency ablation of painful metastases involving bone: a multicenter study. J Clin Oncol.

[12] Dupuy DE, Liu D, Hartfeil D, Hanna L, Blume JD, Ahrar K, Lopez R, Safran H, DiPetrillo T (2010). Percutaneous radiofrequency ablation of painful osseous metastases: a multicenter American College of Radiology Imaging Network trial. Cancer-Am Cancer Soc.

[13] Langley SE, Laing R (2002). Prostate brachytherapy has come of age: a review of the technique and results. Bju Int.

[14] Roos DE, Turner SL, O’Brien PC, Smith JG, Spry NA, Burmeister BH, Hoskin PJ, Ball DL (2005). Randomized trial of 8 Gy in 1 versus 20 Gy in 5 fractions of radiotherapy for neuropathic pain due to bone metastases (Trans-Tasman Radiation Oncology Group, TROG 96.05). Radiother Oncol.

[15] Kaasa S, Brenne E, Lund JA, Fayers P, Falkmer U, Holmberg M, Lagerlund M, Bruland O (2006). Prospective randomised multicenter trial on single fraction radiotherapy (8 Gy x 1) versus multiple fractions (3 Gy x 10) in the treatment of painful bone metastases. Radiother Oncol.

[16] Amouzegar-Hashemi F, Behrouzi H, Kazemian A, Zarpak B, Haddad P (2008). Single versus multiple fractions of palliative radiotherapy for bone metastases: a randomized clinical trial in Iranian patients. Curr Oncol.

[17] Steenland E, Leer JW, van Houwelingen H, Post WJ, van den Hout WB, Kievit J, de Haes H, Martijn H, Oei B, Vonk E, van der Steen-Banasik E, Wiggenraad RG, Hoogenhout J, Warlam-Rodenhuis C, van Tienhoven G, Wanders R (1999). The effect of a single fraction compared to multiple fractions on painful bone metastases: a global analysis of the Dutch Bone Metastasis Study. Radiother Oncol.

[18] van der Linden YM, Lok JJ, Steenland E, Martijn H, van Houwelingen H, Marijnen CA, Leer JW (2004). Single fraction radiotherapy is efficacious: a further analysis of the Dutch Bone Metastasis Study controlling for the influence of retreatment. Int J Radiat Oncol Biol Phys.

[19] Gaze MN, Kelly CG, Kerr GR, Cull A, Cowie VJ, Gregor A, Howard GC, Rodger A (1997). Pain relief and quality of life following radiotherapy for bone metastases: a randomised trial of two fractionation schedules. Radiother Oncol.

[20] Chow E, Hoskin P, Mitera G, Zeng L, Lutz S, Roos D, Hahn C, van der Linden Y, Hartsell W, Kumar E (2012). Update of the international consensus on palliative radiotherapy endpoints for future clinical trials in bone metastases. Int J Radiat Oncol Biol Phys.

[21] Xiang Z, Mo Z, Li G, Gilani S, Zhong Z, Zhang T, Zhang F, Gao F (2016). ^125^I brachytherapy in the palliation of painful bone metastases from lung cancer after failure or rejection of conventional treatments. Oncotarget.

[22] Nath R, Anderson LL, Luxton G, Weaver KA, Williamson JF, Meigooni AS (1995). Dosimetry of interstitial brachytherapy sources. Recommendations of the AAPM Radiation Committee Task Group No. 43. American Association of Physicists in Medicine. Med Phys.

[23] Pignol JP, Rakovitch E, Keller BM, Sankreacha R, Chartier C (2009). Tolerance and acceptance results of a palladium-103 permanent breast seed implant Phase I/II study. Int J Radiat Oncol Biol Phys.

[24] Pereira J, Lawlor P, Vigano A, Dorgan M, Bruera E (2001). Equianalgesic dose ratios for opioids. a critical review and proposals for long-term dosing. J Pain Symptom Manage.

